# Design, Development, and Multi-Characterization of an Integrated Clinical Transrectal Ultrasound and Photoacoustic Device for Human Prostate Imaging

**DOI:** 10.3390/diagnostics10080566

**Published:** 2020-08-07

**Authors:** Sumit Agrawal, Kerrick Johnstonbaugh, Joseph Y. Clark, Jay D. Raman, Xueding Wang, Sri-Rajasekhar Kothapalli

**Affiliations:** 1 Department of Biomedical Engineering, Pennsylvania State University, University Park, State College, PA 16802, USA; sua347@psu.edu (S.A.); kfj5051@psu.edu (K.J.); 2 Penn State Cancer Institute, Pennsylvania State University, Hershey, PA 17033, USA; jclark13@pennstatehealth.psu.edu (J.Y.C.); jraman@pennstatehealth.psu.edu (J.D.R.); 3 Division of Urology, Penn State Milton S Hershey Medical Center, Hershey, PA 17033, USA; 4 Department of Biomedical Engineering, University of Michigan, Ann Arbor, MI 48109, USA; xdwang@umich.edu; 5 Graduate Program in Acoustics, Pennsylvania State University, University Park, PA 16802, USA

**Keywords:** transrectal ultrasound (TRUS), photoacoustic imaging (PAI), prostate cancer (PCa) diagnosis, clinical device

## Abstract

The standard diagnostic procedure for prostate cancer (PCa) is transrectal ultrasound (TRUS)-guided needle biopsy. However, due to the low sensitivity of TRUS to cancerous tissue in the prostate, small yet clinically significant tumors can be missed. Magnetic resonance imaging (MRI) with TRUS fusion biopsy has recently been introduced as a way to improve the identification of clinically significant PCa in men. However, the spatial errors in coregistering the preprocedural MRI with the real-time TRUS causes false negatives. A real-time and intraprocedural imaging modality that can sensitively detect PCa tumors and, more importantly, differentiate aggressive from nonaggressive tumors could largely improve the guidance of biopsy sampling to improve diagnostic accuracy and patient risk stratification. In this work, we seek to fill this long-standing gap in clinical diagnosis of PCa via the development of a dual-modality imaging device that integrates the emerging photoacoustic imaging (PAI) technique with the established TRUS for improved guidance of PCa needle biopsy. Unlike previously published studies on the integration of TRUS with PAI capabilities, this work introduces a novel approach for integrating a focused light delivery mechanism with a clinical-grade commercial TRUS probe, while assuring much-needed ease of operation in the transrectal space. We further present the clinical potential of our device by (i) performing rigorous characterization studies, (ii) examining the acoustic and optical safety parameters for human prostate imaging, and (iii) demonstrating the structural and functional imaging capabilities using deep-tissue-mimicking phantoms. Our TRUSPA experimental studies demonstrated a field-of-view in the range of 130 to 150 degrees and spatial resolutions in the range of 300 μm to 400 μm at a soft tissue imaging depth of 5 cm.

## 1. Introduction

Prostate cancer (PCa) has become the most commonly diagnosed nonskin cancer in American men, with an annual incidence rate of over 170,000 cases [1]. PCa has a relatively low progression rate for patients with early diagnosis (with a five-year survival rate close to 100%). Yet, the survival rate decreases significantly once the cancer has metastasized [2,3]. Therefore, differentiating aggressive from indolent PCa is critical for improving patient outcomes and preventing metastasis and death.

Currently, transrectal ultrasound (TRUS)-guided biopsy is the standard procedure for evaluating the presence and aggressiveness of PCa. Since ultrasound (US) has limited sensitivity to cancerous tissue in the prostate, TRUS-guided biopsies yield 20–30% false negative rates [4,5,6]. A preprocedure magnetic resonance imaging (MRI) and TRUS fusion biopsy has been introduced recently to improve the identification of clinically significant PCa in men with prior negative biopsies [7,8,9,10,11]. However, the spatial errors in coregistering the preprocedural MRI with the real-time TRUS still yields a 10–20% false negative rate [9,10,11].

To reliably differentiate aggressive PCa tumors from indolent PCa and other benign prostate conditions, the imaging technology should be sensitive to unique pathological hallmarks and/or molecular biomarkers of PCa. Towards this goal, researchers have explored several in vitro diagnostic [12,13] and in vivo imaging technologies, such as elastography [14], hyperpolarized ^13^C MRI for mapping metabolic changes associated with PCa [15], and positron emission tomography (PET) with PCa biomarker-specific radiotracers [16]. However, the high cost, limited availability, and use of ionizing radiation (in the case of PET) render these modalities unsuitable for frequent screening, monitoring, or real-time biopsy guidance. 

Alternatively, photoacoustic imaging (PAI) demonstrated the potential to image the functional and molecular contrast of the prostate cancer in preclinical models [17]. Like ultrasonography, PAI relies on the detection of acoustic waves; hence, it is natural to integrate PAI with TRUS to provide complementary optical contrast of deep tissue (up to 12 cm) with sub-mm resolution [18]. The strong intrinsic optical absorption of oxyhemoglobin (HbO_2_) and deoxyhemoglobin (Hb) enables label-free imaging of individual blood vessels, facilitating the detection of angiogenesis, tissue blood volume, and blood oxygen saturation. Furthermore, the sensitivity and specificity of PAI can be enhanced by labeling tumor cells with exogenous contrast agents [19,20] 

The feasibility of PAI of large-animal prostate was first demonstrated in 2010 by Wang et al., by invasively inserting a light guide and a linear US probe (separately) inside a canine abdomen through a lower midline vertical incision [21]. Bell et al. in 2014 demonstrated in vivo canine prostate photoacoustic imaging with transurethral light delivery and a transrectal US probe [22]. In both of these studies, the optical source (light guide) and the ultrasonic detection (transducer) paths were not truly integrated to a clinical TRUS device, and hence, translation to clinics was not feasible. 

Several groups started exploring the integration of optical excitation with the clinical TRUS device to facilitate smooth, safe transrectal photoacoustic imaging of the prostate. Ishihara et al. developed a handheld PAI device by integrating the light guide to a conventional curvilinear TRUS probe [23]. However, the light guide/aperture was simply positioned close to the distal end of the ultrasound array without optimally focusing the light, leading to reduced efficiency of light delivery to the targeted prostatic regions. Another such study presented by Liu et al. in 2019 utilized a glass mirror on both sides of a curvilinear TRUS device to reflect light towards the imaging plane [24]. The placement of mirrors on either side of the curvilinear US array led to a standoff of about 6–8 mm between the array and the tissue, making the overall design less comfortable for transrectal insertion. Moreover, both these studies used single-wavelength excitation for photoacoustic acquisition.

Recently, Kothapalli et al. developed and validated a (first-in-human) transrectal ultrasound and photoacoustic (TRUSPA) device using a custom fabricated 64-element linear capacitive micromachined ultrasound transducer (CMUT) array [25]. The device is side-looking, providing only a sagittal view of the prostate, with customized optical components for bending the light in the ultrasound plane. Some other limitations include a limited field-of-view due to the smaller CMUT array aperture size (20 mm) and lower US image contrast compared to conventional TRUS devices. For the space-constrained TRUSPA imaging, a curvilinear ultrasound array consisting of 128 or 196 elements is desired to generate prostate images with higher quality and a wider field-of-view, for example in conventional TRUS prostate imaging. Although this and previous studies demonstrated the high PA sensitivity of CMUTs [26,27], the unavailability of curvilinear CMUT arrays limit their use for developing a wide field-of-view clinical-grade TRUSPA device. This limitation also extends for emerging piezoelectric micromachined ultrasound transducer (PMUT) arrays, which recently showed good experimental results for deep-tissue photoacoustic imaging applications [28,29]. Further, none of the above TRUS combined photoacoustic studies reported a detailed evaluation of safety parameters, especially when involving customized ultrasound research data acquisition platforms, such as the commonly used Verasonics platforms. 

In this work, we have addressed the above challenges and developed a second-generation handheld integrated TRUSPA device. For this purpose, a clinical-grade, forward-looking 128-element curvilinear TRUS device is integrated with optical components facilitating focused illumination for photoacoustic imaging of functional and molecular information of deep-tissue prostatic regions. We have performed a rigorous characterization of the device and examined the acoustic and optical safety parameters for human prostate imaging. Dual modality imaging capabilities were assessed using experiments on tissue-mimicking phantoms. The rest of the paper is organized as follows. Section 2 presents the design and development of the proposed device. Characterization of imaging performance, safety evaluation, and validation over tissue-mimicking phantoms are presented in Section 3. We conclude the work and propose future directions in Section 4.

## 2. Materials and Methods

In this section, a detailed description of the design and development of the proposed dual-modality transrectal ultrasound and photoacoustic (TRUSPA) device is presented. 

### 2.1. Design and Development of TRUSPA Device 

**Figure 1:**
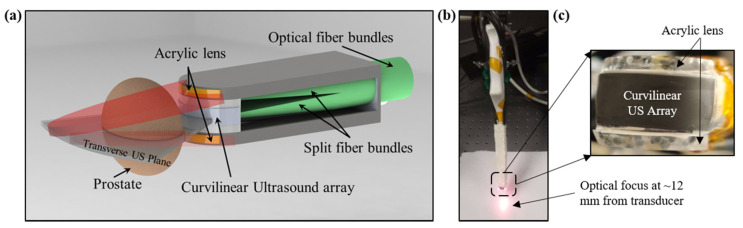
Design of the transrectal ultrasound and photoacoustic (TRUSPA) imaging device. (**a**) Schematic of distal end of the TRUSPA device showing the key components. An optical fiber bundle split into twenty fibers (ten each side) is closely integrated with a commercial curvilinear TRUS probe. Acrylic lenses attached to each side of the probe bend light into the US plane. (**b**) A photograph of the integrated device with optical illumination at 690 nm showing the optical focus at ~12 mm achieved using the acrylic lenses. (**c**) A zoomed view of the distal end of the device with curvilinear US array and the two acrylic lenses attached on both sides for the refraction of light.

A schematic of the proposed TRUSPA device design is shown in Figure 1a. A commercial 128-element curvilinear TRUS probe (C8-4V, ATL, with 8 to 4 MHz extended operating frequency range, 11 mm radius of curvature, 135° field-of-view (FOV)) was adapted for TRUSPA imaging. The outer casing of the probe is shaved from both the sides to develop sufficient space for the integration of optical illumination. Light from a portable laser is coupled to a 2 m long optical fiber bundle with a 6.5 mm diameter fused end, custom-designed by Fiberoptics Systems Inc. The distal end of this optical fiber bundle is split into twenty smaller fibers each with a 1.45 mm inner diameter and 0.55 numerical aperture. Ten of the twenty fibers are separated and attached uniformly to each side of the probe. An acrylic lens sits in front of these ten fibers on each side of the probe to bend the light (approximately 60 degrees) towards the US plane. The two light planes meet at a focal spot ~12 mm from the curvilinear transducer, creating a dark field illumination for deep prostate imaging. The dark-field illumination helps to avoid strong PA signals coming from the top surface of the prostate. Hardened clay is used to provide a smooth finish to the device. The total thickness of the probe after optical fiber integration is limited to 22 mm, allowing relatively easy operation in the transrectal space. Figure 1b shows the device with an optical focus shown ~12 mm from the transducer. A zoomed image of the distal end of the device is shown in Figure 1c, where two acrylic lenses are visible on the top and bottom surfaces of the curviliear US transducer array.

### 2.2. TRUSPA Imaging System: Experimental Setup

**Figure 2:**
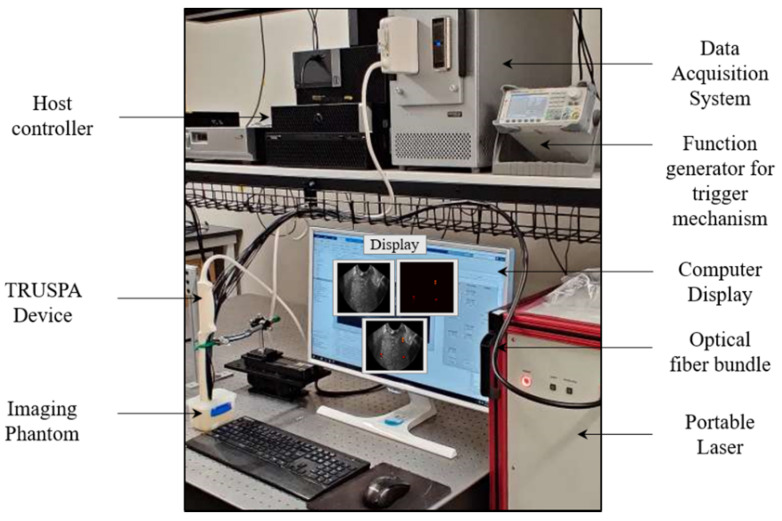
Description of the TRUSPA imaging system. The TRUSPA system consists of following key components. A portable laser: the output of this tunable laser (Phocus Mobile, Opotek Inc., Carlsbad, CA, USA) is coupled to the input end of the optical fiber bundle. Data acquisition system (DAQ): A Vantage 256 (Verasonics, Inc., Kirkland, WA, USA) system is employed for carrying RF signals to and from the TRUSPA device, enabling the real-time acquisition and beamforming of B-mode ultrasound (US) and photoacoustic (PA) images. Function generator: synchronizes the laser with the DAQ system, enabling registration of B-mode US with PA images. Imaging phantom: a tissue-mimicking phantom designed to study the deep-tissue imaging capabilities of the TRUSPA device. Computer display: displays B-mode US (grayscale), PA (red color scale), and coregistered US + PA (overlaid red PA on gray US).

Figure 2 shows the overall TRUSPA experimental setup explaining the key acoustic and optical components and the communication with the data-acquisition hardware. A tunable (in 690–970 nm range), portable optical parametric oscillator (OPO) laser (Phocus Mobile, Opotek, Inc., Carlsbad, CA, USA) source with 10 ns pulse width, 10 Hz pulse repetition frequency and an output energy of 120 mJ per pulse at 730 nm is used to provide the optical illumination. The light from the laser is coupled to the proximal end of the custom-designed optical fiber bundle. The distal end of the optical fiber bundle is integrated to the ultrasound probe, as discussed in Section 2.1 and shown in Figure 1. AC electrical pulses from a data-acquisition (DAQ) hardware (Vantage 256, Verasonics, Inc., Kirkland, WA, USA) are supplied to the curvilinear transducer array of the TRUSPA device for US imaging. The received radio frequency (RF) US and PA signals from the TRUSPA device are transferred to the DAQ for real-time beamforming and display of the B-mode US (with 30 Hz frame rate), PA (10 Hz frame rate), and a coregistered US + PA image, as shown in Figure 2 and supplementary Video S1 (Supplementary Video.mov). A function generator is used to synchronize both the DAQ and the laser illumination by setting the required time delays and thus allowing a proper interleaved, coregistered US + PA image formation.

## 3. Experiments and Results

In this section, an extensive evaluation of the TRUSPA device with the help of (i) rigorous characterization experiments, (ii) examination of the safety parameters for human imaging, and (iii) validations of structural and functional imaging capabilities using several tissue-mimicking phantoms have been presented.

### 3.1. Characterization: Evaluation of 128-Element Curviliear Transducer Array 

**Figure 3:**
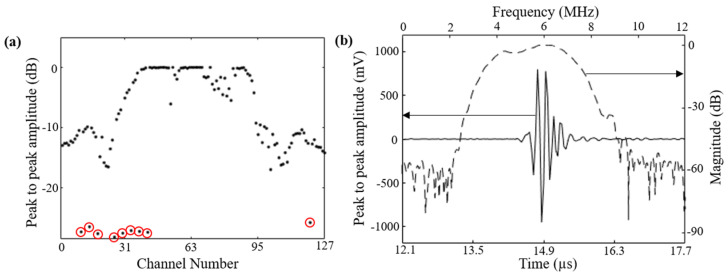
Characterization of the 128-element curvilinear array transducer of the TRUSPA device. (**a**) Shows the peak-to-peak amplitude (in dB) of the first acoustic reflection from a metal slab target placed in front of the curvilinear array (~1.1 cm), plotted for all 128 channels. Poor output from 9 elements, circled in red, indicate that these 9 elements most likely lost connection during the device integration process. (**b**) Shows the pulse-echo waveform measured by the center channel (channel #64) of the array and the corresponding frequency spectrum drawn with a dashed line in dB scale.

Several characterizations were performed to evaluate the performance and sensitivity of the curvilinear ultrasound array. Characterization of the pulse-echo response was achieved by placing a metal slab target in front of the array, perpendicular to the center element at approximately 11 mm distance. The peak-to-peak amplitudes (in dB) of the signals received by each of the 128 elements are plotted in Figure 3a. The nine red-circled measurements in the plot correspond to the possible dead elements in the array. To estimate the frequency and bandwidth of the array, the Fourier-transform of the pulse-echo waveform for the center element (channel #64) was calculated. Figure 3b shows the frequency band shape and the pulse-echo waveform for the center element of the array. The array exhibited a center frequency of 6 MHz and a 6-dB fractional bandwidth of 55%.

### 3.2. Characterization: Ultrasound Field Characterizations 

**Figure 4:**
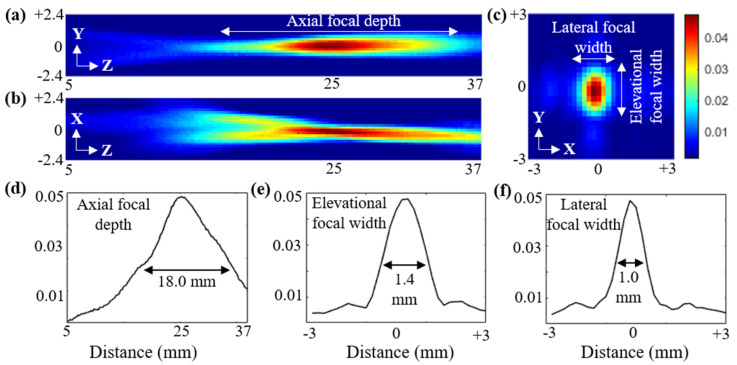
Characterization of the TRUSPA device US field across the elevational (Y–Z, shown in **a**), axial (X–Z, shown in **b**), and lateral (X–Y, shown in **c**) planes, measured under water via hydrophone. (**d**) Beam profile plotted along the axial (Z) direction in the Y–Z field in (**a**), representing the calculated axial focal depth of 18 mm at 50% drop. (**e**,**f**) Beam profiles plotted along the elevational and lateral directions over the focal area of the X–Y field in (**c**), showing the measured elevational and lateral focal width of 1.4 mm and 1.0 mm at 50% drop, respectively.

After characterizing the sensitivity of individual elements in the curvilinear transducer array, characterization of the ultrasound field of the complete array was performed using a calibrated hydrophone (Onda HNP-0400, Onda Corporation, Sunnyvale, CA, USA). The US field plots in all three planes crossing the on-axis focal spot (at ~25 mm distance perpendicular to the center element) were acquired. Figure 4a–c show the output US beam profile of the TRUSPA device, recorded by hydrophone along the elevational (Y–Z), axial (X–Z), and the lateral (X–Y) planes, respectively. The device was placed in a water tank, and the hydrophone was mechanically scanned using an XYZ scanner. The excitation with 1.6 V AC supply yielded ~0.5 MPa output peak-to-peak pressure at the focus (z = 25 mm). The X–Y plane was measured at a distance of 25 mm from the probe. To calculate the focal depth of the TRUSPA device, the beam profile along the axial direction in the Y–Z field was plotted (Figure 4d). The calculated axial focal depth at 50% drop is 18.0 mm. Further, the beam profiles along the elevational and lateral directions in the X–Y field were plotted to measure the focal width in the lateral plane around the focal region. The calculated values of elevational focal width (Figure 4e) and lateral focal width (Figure 4f) at 50% drop are 1.4 mm and 1.0 mm, respectively. 

### 3.3. Evaluation of Safety Parameters

**Figure 5:**
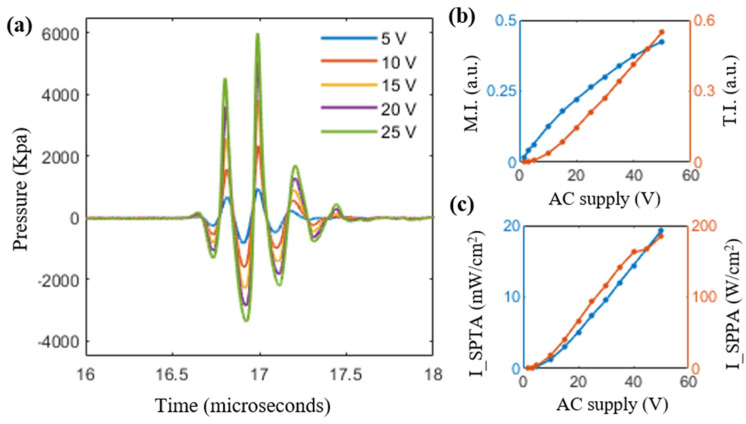
(**a**) Output pressure of the TRUSPA device recorded by hydrophone as a function of varying supply AC voltages in the range of 5 V to 25 V with 5 V intervals. (**b**) Plot of mechanical index (MI) and thermal index (TI) after 0.3 dB.cm^−1^.MHz^−1^ de-ration with respect to the supplied AC voltage. (**c**) Plot of spatial-peak temporal average (I_SPTA_) and spatial-peak pulse average (I_SPPA_) values after 0.3 dB.cm^−1^.MHz^−1^ de-ration with respect to the supplied AC voltage.

In this subsection, an extensive evaluation of the safety parameters for the developed TRUSPA device for human imaging is presented. For the calculation of all the safety parameters, a calibrated hydrophone (Onda HNP-0400, Onda Corporation, Sunnyvale, CA, USA) was placed in front of the TRUSPA device immersed in water. At the focal spot (25 mm perpendicular distance from the center element of the transducer), the peak-to-peak amplitude and the corresponding acoustic pressure (*P_i_*) generated by varying the supplied AC voltage in the range of 1.6 V to 50 V from the Verasonics DAQ was measured. Figure 5 shows the overlaid plots of five representative acoustic pressure waveforms measured by the hydrophone over the supply voltage range of 5 V to 25 V with an interval of 5 V. 

The mechanical index (MI) indicates the likelihood of nonthermal bioeffects, such as cavitation, caused by ultrasonic exposure [30]. In order to assess the risk of exposure, the MI values at the focal spot corresponding to each supply voltage are calculated by dividing the peak negative acoustic pressure (in MPa) with the square root of the center frequency (in MHz), calculated in Section 3.1. These measurements were made under water immersion, which is assumed here to exhibit negligible acoustic attenuation. In order to account for the acoustic attenuation of human tissue, all the measured values of peak negative acoustic pressure were de-rated by 0.3 dB.cm^−1^.MHz^−1^. The obtained de-rated MI values are shown in Table 1. The safety limit set by the Food and Drug Administration (FDA) [31] allows a maximum MI of 1.9 for the acoustic exposure of human tissue. The MI values for the TRUSPA device fall well within the safety limits for supply voltages up to 50 V, indicating the safety of the device for prostate imaging.

Further, two more important measures to evaluate the acoustic exposure safety of a given acoustic pulse are: (i) spatial-peak temporal average (I_SPTA_) and (ii) spatial-peak pulse average (I_SPPA_). The spatial-peak temporal average measures the average intensity during the entire exposure. It represents the overall tissue heating. The spatial-peak pulse average represents the average intensity over a single pulse and thus provides an estimate of the short-term effects of acoustic exposure on biological tissue. Equations (1) and (2) below present the formulas for calculating the *I_SPTA_* and I_SPPA_ values for a given acoustic exposure [32]:

(1) $$I_{SPTA}=PI*PRF$$

(2) $$I_{SPPA}=\frac{PI}{PD}$$

Here, PRF represents pulse repetition frequency and PD represents the pulse duration. The pulse intensity integral (*PI*) is calculated by integrating the intensity of a pulse *(I*) over its duration, as shown in Equation (3) below [32]:

(3) $$PI=\int_{t1}^{t2}I\;dt$$

where the intensity of pulse (*I*) can be calculated by dividing the square of the acoustic pressure by the product of the acoustic speed (*c*) and acoustic density (*d*) of the medium, as shown in Equation (4) below [32]:

(4) $$I=\frac{P_{i}{}^{2}}{cd}$$

The *I_SPTA_* and I_SPPA_ parameters over AC supply voltages from 1.6 V to 50 V were measured and de-rated by 0.3 dB.cm^−1^.MHz^−1^ to account for human-tissue acoustic attenuation. The de-rated values calculated are as shown in Table 1. The FDA safety limit allows a maximum *I_SPTA_* value of 720 mW/cm^2^ and a maximum I_SPPA_ value of 190 W/cm^2^ for the acoustic exposure of human tissue [31]. As shown, at all the supply voltages, both the *I_SPTA_* and I_SPPA_ of the TRUSPA device fall well within FDA safety limits.

Another important acoustic exposure safety parameter is the thermal index (TI). While the MI relates to the risk of mechanical bioeffects, such as cavitation, the TI parameter indicates the risk of thermal bioeffects [33]. TI is calculated by dividing acoustic power at the focal point by the amount of power required to raise the tissue temperature by one degree Celsius [33]. The FDA limit [33] on TI for soft tissue is 6. The TI values obtained over the AC supply voltage range of 1.6 V to 50 V are reported in Table 1 and indicate the safety of the TRUSPA device for human prostate imaging.

### 3.4. Optical Fluence and PAI Field-of-View Characterization

In contrast to ultrasonography, acoustic waves are generated in photoacoustic imaging via the delivery of optical energy to tissue structures with high optical absorbance. As such, delivery of light to deep tissue remains a fundamental challenge in PAI systems, especially for in vivo applications, where tissue typically exhibits high optical scattering. To avoid strong PA signals from the tissue surface, the TRUSPA device was designed with dark field light illumination, with a beam size of approximately 10 mm × 20 mm in air, measured ~12 mm from the distal end of the probe. The measured optical fluence (energy density) is ~10 mJ/cm^2^ at 800 nm, well under the American National Standards Institute (ANSI) maximum permissible exposure limit [34] of 20 mJ/cm^2^. 

**Figure 6:**
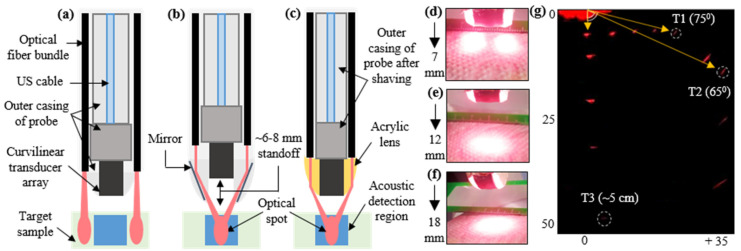
Optical fluence and PAI field-of-view characterization. (**a**) Shows a conventional approach for integrating an optical source with a TRUS probe with no light-bending mechanism. (**b**) Shows another conventional method adopted for reflecting the optical beams around the TRUS probe using two mirrors, leading to a stand-off of ~6–8 mm. (**c**) Shows the obtained optical path with the novel proposed design using two acrylic lenses integrated with the probe, leading to an optical focal spot at ~12 mm and eliminating the stand-off problem. (**d**–**f**) Show the optical fluence profile images captured experimentally with the novel TRUSPA device at ~7 mm, ~12 mm, and ~18 mm distance from the surface of the probe with 690 nm optical wavelength excitation. (**g**) Photoacoustic field-of-view (FOV) measurements made with a 0.3 mm pencil lead target in 20 cm^−1^ reduced optical scattering medium.

Next, the optical source integration of the novel TRUSPA device is compared to that of previous TRUSPA designs. Figure 6a shows a straightforward approach to optical source integration with a curvilinear TRUS probe. Here, two optical fiber bundles are attached parallel to the outer casing of the probe. As shown, the ultrasound detection plane and the obtained optical foci are misaligned when observed over the target sample placed approximately an inch from the surface of the device. This results in an optical blind spot in the US imaging plane, causing diminished PAI resolution and contrast. To overcome this, recent designs [24] have proposed the integration of two optical mirrors at the distal end of the probe, as shown in Figure 6b. These mirrors reflect light toward the US detection area, and thus an optical focus can be obtained in the transverse US plane. However, the placement of these mirrors close to the transducer, as shown, leads to (i) a standoff region of about 6–8 mm labelled in Figure 6b and (ii) a severe problem with the geometry of the probe that prohibits ease of operation in the transrectal space. Overcoming these limitations, the design proposed here integrates the optical path with the TRUS probe by following the schematic in Figure 6c. The outer casing of the probe is shaved approximately 5 mm on each side to reduce the total thickness. The twenty smaller fibers of the optical fiber bundle (as described in Section 2.1) are then split into groups of ten and attached to each side of the probe. The light from these fibers is bent using the two acrylic lenses attached to the distal end of the probe, as shown. These lenses bend the light by ~60 degrees and result in an optical focus ~12 mm from the transducer surface in the US imaging plane. This design not only optimizes the fluence profile for prostate imaging but also provides a smooth finish to the probe, enabling easier operation in transrectal imaging scenarios. 

The optical fluence profile obtained from the novel design can be visualized with the help of the three representative images shown in Figure 6d–f at a distance of ~7 mm, ~12 mm, and ~18 mm from the surface of the probe, respectively, at 690 nm excitation wavelength. As shown, the two beams coming from the sides of the probe are separate when visualized ~7 mm from the probe. They coincide with each other to form a proper optical focus at ~12 mm. Beyond the focal spot, the beam diameter is slightly increased when observed at ~18 mm.

To compare the photoacoustic FOV of the novel TRUSPA device with previously reported devices, a 0.3 mm pencil lead target was placed orthogonal to the imaging plane at various positions in an optical scattering medium (20 cm^−1^ reduced scattering coefficient). As can be seen in Figure 6g, the target T1 (~75 degrees off from the center of the transducer) suggests an FOV of ~150 degrees at a depth of ~7 mm. At ~15 mm depth, the target T2 suggests an FOV of ~130 degrees. This matches the US FOV (~135 degrees) reported in the datasheet of the C8-4v probe, as well as the PA FOVs reported for the designs in Figure 6a (~160 degrees [23]) and Figure 6b (~145 degrees [24]). The target (T3) in Figure 6g also demonstrates the capability of the TRUSPA device to image PA targets up to 5 cm deep in a highly scattering medium.

### 3.5. Structural Imaging Capabilities over a Scattering Phantom:

**Figure 7:**
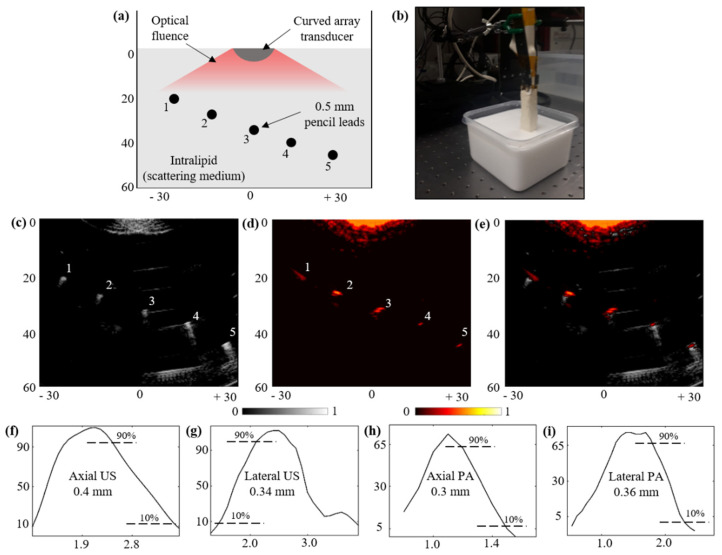
Structural imaging capabilities of the TRUSPA device. (**a**) Shows the schematic of the structural phantom with 5 pencil lead targets of 0.5 mm diameter inside an optically scattering medium with a reduced scattering coefficient of 20 cm^−1^. The curved transducer array of the TRUSPA device and the generated optical fluence are also shown. (**b**) Shows a picture of the imaging setup with the TRUSPA device mounted over a tank with a structural phantom immersed in a scattering intralipid medium. (**c**–**e**) Show the US, PA, and coregistered US + PA B-mode images of the structural phantom imaged with the TRUSPA device. (**f**–**i**) Shows the line spread functions of the US and PA amplitudes for the 3rd pencil lead target in the US and PA images, plotted in the axial and lateral directions, respectively. The calculated resolutions are displayed in the plots. Scale: mm. Colorbar: normalized amplitudes.

To validate the structural imaging capabilities of the novel TRUSPA device, an acrylic tank with five holes was fabricated. Figure 7a shows the positioning of the holes with five pencil lead targets of 0.5 mm diameter inserted into the holes. The experimental setup is shown in Figure 7b. Intralipid was mixed into water to mimic a background scattering coefficient of 20 cm^−1^. The TRUSPA device was mounted on a stage with the structural phantom placed inside the scattering medium. Due to the acoustic impedance mismatch between the pencil lead targets and the medium, all five targets are clearly seen in the US image, as shown in Figure 7c. Due to the high optical absorption of pencil lead compared to the scattering medium, PA signals are generated for all five targets. Figure 7d shows the B-mode PA image generated with the TRUSPA device and Figure 7e shows the coregistered US + PA image of the structural phantom, with US in grayscale and PA overlaid in hot scale. To study the resolution of our device, the peak US and PA amplitudes were plotted over the 3rd pencil lead target in the US and PA images along the axial and lateral directions, as shown in Figure 7f–i. The resolution is calculated using half of the distance between 90% to 10% of the maximum signal amplitudes in the line spread functions. The calculated axial and lateral resolutions for the US image are 0.4 mm and 0.34 mm, respectively. The same calculations for the PA image yield PA axial and lateral resolutions of 0.3 mm and 0.36 mm, respectively. 

### 3.6. Functional Imaging Capabilities with Prostate Tissue-Mimicking Phantom:

**Figure 8:**
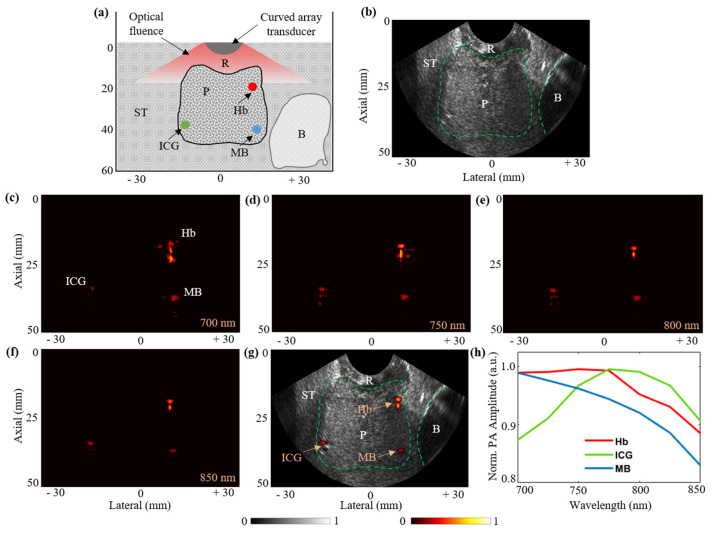
Functional imaging using the TRUSPA device. (**a**) Shows a diagram of the prostate tissue-mimicking phantom with the prostate (P), soft-tissue (ST), bladder (B), and rectum (R) regions highlighted. The three optically transparent tubes with an outer diameter of 0.3 mm filled with bovine blood (Hb), indocyanine green (ICG), and methylene blue (MB) are shown embedded in the prostate region. (**b**) A B-mode US image obtained for the phantom in (**a**) with the TRUSPA device. (**c**–**f**) B-mode PA images obtained for the phantom at wavelengths 700 nm, 750 nm, 800 nm, and 850 nm, respectively. (**g**) Coregistered B-mode US + PA image with anatomical (US) information displayed in grayscale and the molecular information (PA) displayed in hot color scale. (**h**) PA spectral plots obtained for the three molecular target regions.

In this subsection, the functional imaging capabilities of the TRUSPA device are evaluated. A prostate tissue-mimicking phantom was fabricated, as shown in Figure 8a. In order to mimic the surrounding soft-tissue (ST) region, 1.5% agarose gel was mixed with (i) an intralipid solution to achieve a reduced optical scattering coefficient of 20 cm^−1^ and (ii) a 3% by weight corn-flour powder to generate increased acoustic heterogeneity. The hypo-echoic bladder (B) region was obtained by embedding a balloon filled with water as shown in the schematic in Figure 8a. Next, to mimic the prostate (P) tissue region, a 2% by weight corn-flour powder was combined with 1.5% agarose solution, thus differentiating the background soft tissue from the prostate region. The concentration of intralipid was maintained similar to the soft-tissue region to have similar optical scattering. The rectum (R) was mimicked with the same solution as the soft tissue and was used as the interface between the TRUSPA device and the phantom. During the process of making the prostate phantom, three 0.3 mm outer-diameter optically transparent tubes were embedded at different depths, as shown in Figure 8a. The tubes were filled with bovine blood (Hb, Bovine Blood CITR, 301 Carolina Biological Supply, Charlotte, NC, USA, placed at 19 mm depth), indocyanine green (ICG, 1 mM concentration, placed at 34 mm depth), and 1 mM methylene blue (MB, 1 mM concentration, placed at 37 mm depth), enabling the evaluation of the functional imaging capabilities of the TRUSPA device.

Next, the results of using the TRUSPA device to image the prostate phantom described above are presented. Figure 8b shows a typical B-mode US frame displaying the anatomical information of the fabricated prostate tissue phantom with the prostate (P), bladder (B), soft-tissue (ST), and rectum (R) regions labelled. Figure 8c–f show the B-mode PA images acquired at 700 nm, 750 nm, 800 nm, and 850 nm optical excitation wavelength, respectively. Thanks to the high optical absorption of the three molecular targets embedded in the prostate region, the PA images are able to display all three molecules with varying PA intensities depending on their optical absorption spectra. Figure 8g shows a coregistered B-mode US + PA image at 850 nm with the US image in grayscale and the PA image overlaid in hot scale. The mean PA intensities for the three target regions as a function of excitation wavelength are plotted in Figure 8h. The obtained plots for the Hb, ICG, and MB targets closely match with their respective standard spectral plots [35]. For example, the peak response of ICG at around 780 nm and the standard decay of the MB response with increasing wavelength can be observed in Figure 8h. These results demonstrate the feasibility of the TRUSPA device for structural and functional imaging of deeper tissue regions.

## 4. Discussion

The novel TRUSPA device described and characterized here for human prostate imaging improves upon previously reported TRUSPA devices by integrating optical fibers and custom-fabricated lenses with a commercially available transrectal ultrasound device. The TRUSPA device is designed such that the optical lenses fit flush with the distal end of the probe, resulting in a smooth finish. The forward-facing curvilinear ultrasound sensor array enables a higher field-of-view compared to our previous clinically tested TRUSPA device [25].

The rigorous characterization of the novel TRUSPA device presented here includes measurements of the emitted optical energy density, acoustic mechanical index, acoustic thermal index, derated I_SPTA_, and derated I_SPPA_. Although each of these measurements as a function of supplied AC voltages up to 50 V (Table 1) fell within the FDA safety limits for ultrasound exposure, it is recommended to operate the devices following the as low as reasonably achievable (ALARA) precautions [36,37]. The split beam on either side of the probe is refracted with custom lenses and converges at a point approximately 12 mm from the probe tip. To ensure optical radiation safety, the optical fluence of the TRUSPA device must be kept below the ANSI safety limit for maximum permissible laser fluence/exposure (MPE) to the skin that is rectal surface for prostate imaging. For example at 800 nm MPE is 31.7 mJ/cm^2^ and the TRUSPA studies in this work were conducted at 10 mJ/cm^2^. Measurement of the curvilinear transducer ultrasound field revealed a relatively narrow focal zone in the lateral and elevational dimensions compared to our previous TRUSPA device [25], due to the higher element count and the curvilinear shape of the US sensor array. 

Imaging experiments with a structural and a prostate-mimicking phantom demonstrated that the novel TRUSPA device is capable of imaging both structural/mechanical contrasts (US) as well as functional/molecular contrasts (PA) with sub-mm spatial resolution. Each of the imaging experiments utilized optically scattering phantoms to produce optical fluence distributions realistic for in vivo tissue imaging. The transrectal prostate-mimicking phantom was designed with realistic density and acoustic heterogeneity to mimic different prostatic regions including the adjacent bladder region. US images of the prostate phantom acquired with the TRUSPA device show clearly the prostate, bladder, and rectum regions, whereas, PA images show three different optically absorbing targets placed within the prostate region. Further, via multispectral PAI with the TRUSPA device, the unique optical absorption spectra of the three targets, hemoglobin, ICG, and methylene blue, were recovered. This demonstrates the potential of the TRUSPA device for functional imaging of angiogenesis, blood-oxygen saturation and high-contrast in vivo imaging of contrast agents, such as ICG. These capabilities will likely help reduce the false-negative rate in prostate cancer detection by providing additional functional information during guided biopsy. The ergonomic integration of optical components with a commercially available TRUS probe makes the TRUSPA device described here ideal for adoption by clinicians practicing already-widespread TRUS-guided prostate biopsies. It is expected that the reported second generation TRUSPA device with improved imaging performance will likely help achieve better clinical results on human prostate imaging studies. 

## 5. Conclusions

A transrectal ultrasound and photoacoustic imaging probe was developed by integrating focused light delivery with a commercial TRUS prostate probe. Characterization of the TRUSPA probe’s optical energy density, mechanical and thermal indices, as well as *I_SPTA_* and I_SPPA_ acoustic intensities indicated that the probe is safe for human imaging for source voltages up to 50 V. The potential of the probe for human prostate imaging was demonstrated by imaging tissue-mimicking phantoms. Multispectral photoacoustic imaging experiments with a prostate-mimicking phantom demonstrated the functional imaging capabilities of the TRUSPA device. High-quality images were obtained at imaging depths up to 5 cm with optical fluence kept well below the ANSI safety limit. The photoacoustic probe remains compact for in vivo imaging, with the compact size and smooth finish making it patient-friendly for clinical transrectal applications. The combination of photoacoustic and ultrasound imaging demonstrated in this study has a potential to enhance the diagnostic accuracy and precise localization of prostate cancer, targeted needle biopsies, and thus help reduce the necessity for painful repeated biopsies and associated high medical costs.

## Figures and Tables

**Table 1:** Mechanical index (MI), spatial-peak temporal average (I_SPTA_), spatial-peak pulse average (I_SPPA_), and thermal index (TI) values after 0.3 dB.cm^−1^.MHz^−1^ de-ration at 2.5 cm depth, measured with respect to the supply AC voltages for the TRUSPA device.

Supplied AC Voltage (Volts)	Peak Negative Voltage (mV)	Peak Negative Pressure (MPa)	De-Rated Pressure (MPa)	Mechanical Index (MI)	I_SPTA_ (mW/cm^2^)	I_SPPA_ (W/cm^2^)	Thermal Index (TI)
1.6	10.7	0.2192	0.0418	0.0170	0.01	0.22	0.0003
3.2	25.1	0.5172	0.0986	0.0402	0.09	1.43	0.0026
5.0	40.0	0.8234	0.1569	0.0641	0.27	3.97	0.0077
10.0	78.2	1.6085	0.3065	0.1251	1.3	18.26	0.0371
15.0	111.7	2.2981	0.4379	0.1788	2.99	40.64	0.0854
20.0	138.9	2.8578	0.5446	0.2223	5.12	66.88	0.1463
25.0	164.3	3.3800	0.6440	0.2629	7.44	93.50	0.2126
30.0	188.6	3.8805	0.7394	0.3019	9.54	116.54	0.2726
35.0	212.0	4.3628	0.8313	0.3394	12.04	141.61	0.3440
40.0	233.0	4.7936	0.9134	0.3729	14.43	162.45	0.4123
45.0	250.2	5.1479	0.9809	0.4005	16.82	167.01	0.4806
50.0	265.3	5.4585	1.0401	0.4246	19.23	184.91	0.54940

## Supplementary Materials

The following are available online at https://www.mdpi.com/2075-4418/10/8/566/s1.

## Abbreviations

PA	Photoacoustic
US	Ultrasound
PAI	Photoacoustic Imaging
PCa	Prostate Cancer
FOV	Field of View
TRUS	Transrectal Ultrasound
TRUSPA	Transrectal Ultrasound and Photoacoustic
